# Editorial: Sexual harassment in the workplace: prevalence, etiologies, prevention and management strategies

**DOI:** 10.3389/fpubh.2023.1332131

**Published:** 2023-11-22

**Authors:** Mahlagha Dehghan, Parvin Mangolian Shahrbabaki

**Affiliations:** Nursing Research Center, Kerman University of Medical Sciences, Kerman, Iran

**Keywords:** sexual harassment, workplace, prevalence, etiologies, prevention, management strategies

In recent decades, there is a high prevalence of sexual harassment and gender discrimination in various professions (1–4). Sexual harassment in the workplace is characterized by repetitive and unwelcome sexual behaviors, encompassing verbal, physical, psychological, and visual forms. These behaviors are often accompanied by acts of humiliation, insults, or threats that jeopardize the wellbeing of the targets (1, 5). The consequences of such occurrences can be severe, resulting in physical, psychological, and emotional harm, as well as difficulties in the workplace. This may manifest as resignations, frequent absences, strained relationships with colleagues, counterproductive work behaviors, and a decline in energy, work efficiency, creativity, and overall job satisfaction (5–9).

The prevalence of sexual harassment has been the subject of numerous studies, revealing a wide range of findings that vary across different countries due to their unique cultural characteristics. This diversity makes it challenging to establish a unified international perspective on workplace sexual harassment. However, it is possible to share common experiences and preventive strategies that can be beneficial for societies in different countries. These strategies can aid in the identification, management, and monitoring of such behavior and its impact on targets. An important aspect that underscores the significance of addressing workplace sexual harassment is the underreporting of incidents. Many cases go unreported due to various factors, including cultural influences, fear of negative consequences, peer pressure, and the reluctance of targets (5, 8, 9). To address this issue, a Research Topic was proposed to gather manuscripts focusing on recent advancements and future perspectives regarding sexual harassment in the workplace. The aim was to facilitate the development of effective prevention measures in diverse work environments, explore legal and ethical strategies for managing post-sexual harassment issues, and enhance workplace safety. In conclusion, this Research Topic received four accepted papers, comprising one brief research report and three original research articles. These contributions contribute to the ongoing efforts to combat workplace sexual harassment and promote a safer and more inclusive work environment.

In the study conducted by Hardies, it was found that a significant number of employees in global accounting and law firms in Belgium experienced some form of sexual harassment in the workplace. Specifically, 88.5% of women and 83.3% of men reported experiencing sexual harassment at least once or twice in the past 24 months, with verbal harassment being the most common form. The study also revealed a positive correlation between workplace sexual harassment and the perceived acceptance of such behavior by peers, as well as a negative correlation with job level (Hardies). Another qualitative study by Zeighami et al. focused on the cultural atmosphere within the workplace and its impact on the non-disclosure of sexual harassment among Iranian nurses. The findings highlighted various barriers that prevent targets from disclosing incidents of sexual harassment. These barriers include fear of social stigmas, organizational and legal obstacles (such as weak legislation, lack of support from authorities, and fear of job instability), family barriers, and personal barriers. The study emphasized the need for policies and strategies to be developed to encourage targets to come forward and disclose instances of sexual harassment (Zeighami et al.). These research findings confirm the connection between sexual harassment and workplace culture. They underscore the importance of implementing policies and strategies that create a supportive environment for targets and encourage them to report incidents of sexual harassment. By addressing these barriers and promoting a culture of accountability, organizations can work toward preventing and addressing sexual harassment in the workplace.

The #MeToo movement on social media has played a significant role in raising awareness about sexual harassment and providing a platform for individuals to share their experiences. Siuta et al. conducted a mixed-methods study to explore the factors influencing targets' decisions to participate (or not) in the #MeToo campaign. Their findings revealed that a history of sexual harassment was the most influential predictor of posting on #MeToo, along with factors such as power dynamics and interpersonal contact. Targets who felt a sense of responsibility to share their stories, sought support, and anticipated emotional benefits were more likely to disclose their experiences. On the other hand, factors such as negative emotions related to the event, concerns about privacy, fear of consequences, and the timing of the event influenced individuals' decisions not to disclose their experiences (Siuta et al.). It is important to note that online disclosure of sexual harassment often reflects the barriers and challenges targets face when considering formal reporting procedures. In his study, Wang examined the legislative and judicial responses to workplace sexual harassment in China. The findings revealed that while China has made commendable progress in establishing an anti-sexual harassment legal system, there are still certain limitations. One of the drawbacks identified is the need for a clearer and more comprehensive definition of sexual harassment, emphasizing that it is a form of gender discrimination that can include hostile environment harassment not directed at a specific individual. Furthermore, the study highlighted the necessity for further delineation of the employer's obligations in preventing and addressing sexual harassment. Currently, there is a lack of guidelines for establishing fair and effective grievance procedures within organizations. Additionally, the difficulty of proving sexual harassment in litigation remains unresolved, posing challenges for targets seeking legal recourse. Another significant issue identified is the lack of adequate legal protection for workers who are not in traditional employment relationships. These individuals often face insufficient safeguards against work-related sexual harassment. Overall, Wang's study underscores the need for improvements in China's anti-sexual harassment legal framework, including clearer definitions, enhanced employer obligations, guidelines for grievance procedures, and increased protection for workers in non-traditional employment arrangements.

In summary, multiple studies within this Research Topic have highlighted the ongoing prevalence of sexual harassment in the workplace, affecting both genders. One significant contributing factor to this issue is the presence of barriers that hinder targets from disclosing instances of sexual harassment. With the increasing use of social media as a platform for disclosure, these findings suggest that organizations should acknowledge the validity of online reports of sexual harassment and consider the limitations of their formal reporting systems as potential reasons for individuals resorting to online disclosure. Further research is needed to better understand the mechanisms of disclosing sexual harassment and to develop strategies that mitigate the potential negative consequences for targets. By addressing these issues, organizations can work toward effectively reducing the occurrence of sexual harassment in the workplace.

## Author contributions

MD: Conceptualization, Data curation, Writing—original draft, Writing—review & editing. PM: Writing—review & editing.
